# Highlights of glycosylation and adhesion related genes involved in myogenesis

**DOI:** 10.1186/1471-2164-15-621

**Published:** 2014-07-22

**Authors:** Vincent Grassot, Anne Da Silva, James Saliba, Abderrahman Maftah, Fabrice Dupuy, Jean-Michel Petit

**Affiliations:** 1 INRA, UMR 1061 Unité de Génétique Moléculaire Animale, Université de Limoges, Faculté des Sciences et Techniques, 123 Avenue A. Thomas, Limoges 87060, France

**Keywords:** Glycosylation related genes, Satellite cells, C2C12, Myogenesis, Adhesion, Integrins, ITGA11, Early adipogenesis, Chst5

## Abstract

**Background:**

Myogenesis is initiated by myoblast differentiation and fusion to form myotubes and muscle fibres. A population of myoblasts, known as satellite cells, is responsible for post-natal growth of muscle and for its regeneration. This differentiation requires many changes in cell behaviour and its surrounding environment. These modifications are tightly regulated over time and can be characterized through the study of changes in gene expression associated with this process. During the initial myogenesis steps, using the myoblast cell line C2C12 as a model, Janot et al. (2009) showed significant variations in expression for genes involved in pathways of glycolipid synthesis. In this study we used murine satellite cells (MSC) and their ability to differentiate into myotubes or early fat storage cells to select glycosylation related genes whose variation of expression is myogenesis specific.

**Results:**

The comparison of variant genes in both MSC differentiation pathways identified 67 genes associated with myogenesis. Comparison with data obtained for C2C12 revealed that only 14 genes had similar expression profiles in both cell types and that 17 genes were specifically regulated in MSC. Results were validated statistically by without *a priori* clustering. Classification according to protein function encoded by these 31 genes showed that the main regulated cellular processes during this differentiation were (i) remodeling of the extracellular matrix, particularly, sulfated structures, (ii) down-regulation of O-mannosyl glycan biosynthesis, and (iii) an increase in adhesion protein expression. A functional study was performed on *Itga11* and *Chst5* encoding two highly up-regulated proteins. The inactivation of *Chst5* by specific shRNA delayed the fusion of MSC. By contrast, the inactivation of *Itga11* by specific shRNA dramatically decreased the fusion ability of MSC. This result was confirmed by neutralization of *Itga11* product by specific antibodies.

**Conclusions:**

Our screening method detected 31 genes specific for myogenic differentiation out of the 383 genes studied. According to their function, interaction networks of the products of these selected genes converged to cell fusion. Functional studies on *Itga11* and *Chst5* demonstrated the robustness of this screening.

## Background

Satellite cells are adult stem cells specific to skeletal muscle. They are located between the basal lamina and striated muscle cells in muscle tissue, and their principal roles are post-natal growth, maintenance and the regeneration of skeletal muscle [1-3]. Satellite cells may undergo asymmetric division for their renewal and produce daughter cells that enter into myogenic differentiation [4]. Satellite cells are multipotent and can differentiate into early fat storage cells or osteoblasts under different environmental conditions [5,6]. Gene expression comparison allows for the characterization of the genes specific to each pathway.

Myogenesis is composed of three steps, (i) alignment of cells, (ii) fusion of cells in myotubes, and (iii) maturation of myotubes. These three steps are regulated by various transcription factors such as the myogenic regulation factors (Mrfs) [4,7-11]. The study of gene expression variation during cell differentiation is fluently used to determine the genes with the most interest [12]. It is also well established that interactions between the extra-cellular matrix and cells [13] as well as cell-cell interactions play a major role in myogenesis [13]. Such regulations and interactions are different from those involved in early-adipogenesis. Therefore, both pathways require tightly regulated recognition systems. One of the better systems to enhance specificity of recognition is glycans and adhesion proteins.

Glycosylation is a process that leads to the formation of a great diversity of glycan structures. These structures are specifically involved in response to the cell environment during developmental stages, and cell fate [14]. We selected genes whose products are involved in glycan synthesis (e.g. nucleotide sugar transporters and glycosyltransferases) as well as genes encoding protein, which recognize glycan structure such as lectins (e.g. selectin), adhesion molecule family (e.g. melanoma cell adhesion molecule) and other adhesion proteins (e.g. integrin family). These genes are called Glycosylation Related Genes (GRG). Janot *et al.* demonstrated the change in expression for some of these genes during early myogenic differentiation of the murine cell line C2C12 [15]. Using this cell model, they suggested that myoblast fusion may require glycosphingolipid rearrangements and/or terminal modifications on glycolipids and glycoproteins (such as fucosylation and sialylation). Among glycoproteins, the adhesion proteins must play a crucial role in cell migration and adhesion; one of the most important families is composed of the integrins [16-18].

Integrins are plasma membrane heterodimers that mediate both cell-cell and cell-extracellular matrix interactions [19]. Integrin subfamilies are classified on the basis of the association of a common β subunit with distinct α subunits to form unique heterodimers. The integrins ITGA4 and ITGB1 have already been described for their myogenic role. They form the VLA-4 complex, an essential adhesion complex interacting with VCAM1 to influence cell alignment and/or cell fusion [20].

In this study, we compared the expression of 383 genes during the differentiation of murine satellite cells (MSC) into myotubes or early fat storage cells. Comparison of gene expressions in both differentiation pathways and previous data on C2C12 [15] revealed that only 31 genes were mainly involved in myogenesis. Fourteen of them have the same variation profile during C2C12 and MSC myogenesis. The remaining seventeen showed a variation only during MSC myogenesis while they were significantly expressed without changes during C2C12 differentiation; e.g. the gene encoding the integrin alpha 11 subunit (*Itga11*). The use of shRNA or neutralizing antibodies against this integrin subunit decreased cell fusion by at least 50%. Thus *Itga11* is critically involved in myotube formation using MSC as a model.

## Results

### MSC differentiation and selection of GRG specific to myogenesis

**Figure 1:**
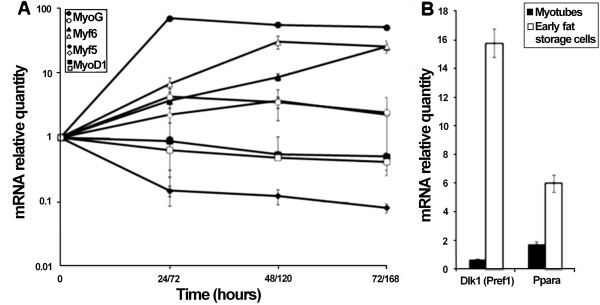
**Expression of myogenic and early step adipogenic markers. A.** The expression of four myogenic regulation factors (*MyoG* (circles), *MyoD1* (squares), *Myf5* (diamonds), *Myf6* (triangles)) during satellite cell differentiation in myotubes (black) and satellite cell trans-differentiation in early fat storage cells (white); standard error was calculated from three independent experimentations. **B.** Relative expression of early adipogenic markers *Ppara* and *Dlk1*, in myotubes (black) and early fat storage cells (white) in comparison with expression in undifferentiated satellite cells, triplicates were used to calculate the standard error.

To identify genes which displayed an expression variation in myogenesis with MSC as progenitor cells, we profiled and compared gene expression during myogenesis or early-adipogenesis. MSC seeded on Matrigel® were differentiated by reduction of serum for 72 hours or trans-differentiated in the presence of ambient 50 mM glucose for 168 hours. The time points were chosen to obtain a percentage of myotubes in myogenic differentiation similar to the percentage of early fat storage cells obtained in trans-differentiation. The differentiation state was confirmed by (i) staining of nuclei in myotubes to quantify the percentage of fusion or to follow fat storage accumulation to determine the trans-differentiation state (Additional file 1); and (ii) measurement of *MyoG*, *MyoD1*, *Myf5* and *Myf6* myogenic markers and *Dlk1* and *Ppara* markers of the early adipogenic step (Figure 1). In myogenic differentiation conditions, *MyoG* expression dramatically increased (70 fold) during the first 24 hours before reaching a plateau (Figure 1) whereas in trans-differentiated MSC it did not exceed 4 fold. A contrasting variation of *Myf5* expression was observed, with expression increasing during myogenesis only. Surprisingly, *Myf6* and *MyoD1* transcription were similar in both pathways. This similarity between trans-differentiation and myogenesis could be explained by the presence of ambient glucose which tends to increase myogenic differentiation in cells already engaged in this process [21]. As expected for the adipogenic markers *Dlk1* and *Ppara,* expression increased notably during trans-differentiation whereas no significant variations were observed during myogenesis (Figure 1B). These results showed the precise control of MSC myogenic differentiation into myotubes or trans-differentiation into early fat storage cells, respectively.

Thus, we can follow and compare the expression of glycogenes and genes encoding adhesion proteins during MSC differentiation in both pathways. Only GRG with a change in mRNA expression of at least two folds was retained for each differentiation pathway. Using this comparative approach we identified 112 genes with a significant variation. Among them, only 67 genes had a variation specific for MSC myogenesis (Additional file 2). The remaining 45, which also varied during early adipogenesis, were discarded. Then, we compared our results with the myoblastic cell line C2C12 [15].

### GRG is specifically involved in myogenesis

**Figure 2:**
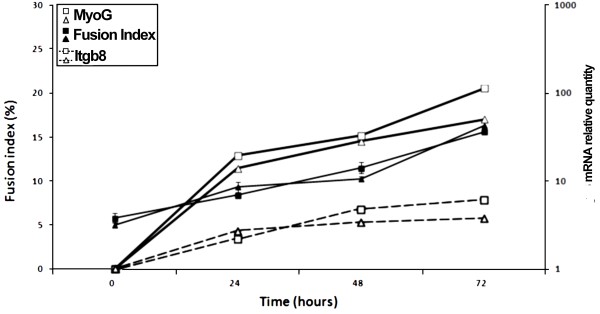
**Myogenic differentiation of C2C12 with or without Matrigel®.** The C2C12 cells were seeded at 5x10^3^ cells/cm^2^ on plates coated (square) or not (triangle) with Matrigel®. The fusion index was measured every 24 hours after differentiation induction by serum starvation (black symbols), vertical bars correspond to standard errors (n = 3). Relative mRNA quantities of *MyoG* (solid line) or *Itgb8* (dotted line) were also measured every 24 hours by qRT-PCR using Taqman probes (white symbols).

Since C2C12 cultures were seeded on plates without Matrigel® in the previous study [15], we controlled that culture on Matrigel® did not improve the differentiation potential of these cells. The comparison between cultures of C2C12 on plates coated with Matrigel® or without it revealed no significant difference in differentiation induction, as recently reported [22]. We also demonstrated that no difference appeared in the fusion index and Myogenin expression (Figure 2). The expression of *Itgb8* as a reference, during myogenic differentiation of C2C12 seeded on plates coated with Matrigel® or not, showed no difference between the two culture conditions (Figure 2).

**Figure 3:**
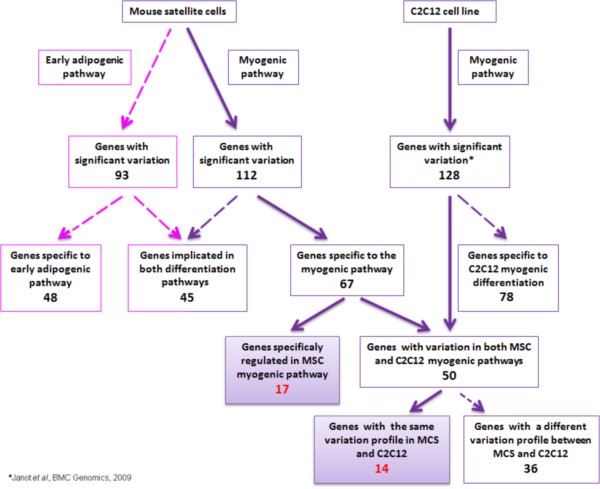
**Determination of myogenesis specific genes.** Comparison between 93 genes significantly regulated in early adipogenic differentiation (pink arrows) and 112 in myogenic differentiation (purple arrows) of Mouse Satellite Cells (MSC) revealed 67 genes specifically regulated during myogenesis. Among them, 17 genes varied only in MSC and 14 were regulated similarly in C2C12 cells (bold red characters). They represent the 31 genes of interest in this study. Dotted arrows correspond to genes discarded during the screening.

The comparison of 67 specifically up or down regulated genes during MSC myogenesis to the results obtained by Janot e*t al.*[15] led to the identification of two subgroups. The first included 17 genes without expression variation throughout C2C12 differentiation whereas their expression varied during MSC differentiation (group B, Table 1). In the second subgroup, 50 genes were identified whose expression varied in both cell types (Figure 3). Among the latter, only 14 had similar variation profiles during C2C12 and MSC myogenesis (group A, Table 1). Therefore, 31 genes seemed to be essential for myogenic differentiation (Figure 3) and were retained for further studies.

**Table 1:** List of 31 selected genes whose expression varied during myogenesis only

			**mRNA Relative quantity according to differentiation time**				
	**Gene symbol**	**Expression pattern**	**0 h**	**12 h**	**24 h**	**48 h**	**72 h**
**A**	*Art1*	Up-regulated	1	1.759	1.205	**13.63**	**39.324**
	*Chst5*	Up-regulated	1	**22.203**	**53.793**	**18.009**	**8.642**
	*Clec2d*	Up-regulated	1	1.463	**2.017**	1.222	**2.065**
	*Galntl1*	Up-regulated	1	1.455	1.635	0.87	**2.689**
	*Gcnt2*	Up-regulated	1	0.84	**2,000**	1.539	1.324
	*Icam2*	Up-regulated	1	**2.037**	**3.854**	**2.465**	**5.674**
	*Itga6*	Up-regulated	1	**3.773**	**2.976**	**2.037**	n.d.
	*Lgals7*	Up-regulated	1	0.468	**2.174**	**2.332**	**4.369**
	*Chst10*	Down-regulated	1	0.641	0.572	**0.455**	0.726
	*Chst8*	Down-regulated	1	**0.204**	**0.18**	**0.087**	**0.069**
	*Clec4d*	Down-regulated	1	1.161	**0.322**	**0.475**	0.913
	*Clgn*	Down-regulated	1	**0.012**	**0.067**	**0.094**	0.549
	*Fut10*	Down-regulated	1	**0.372**	**0.366**	**0.444**	0.566
	*Itgb7*	Down-regulated	1	**0.287**	**0.285**	**0.165**	**0.435**
**B**	*B4galt1*	Up-regulated	1	1.09	1.427	1.613	**2.585**
	*Cd248*	Up-regulated	1	**2.298**	**3.064**	**5.072**	**10.464**
	*Chst12*	Up-regulated	1	0.894	1.238	1.498	**2.177**
	*Cmah*	Up-regulated	1	1.99	**2.451**	**2.611**	**2.394**
	*Fcna*	Up-regulated	1	**5.418**	**2.369**	**2.143**	**2.827**
	*Has1*	Up-regulated	1	**4.283**	0.015	**4.635**	**7.508**
	*Has2*	Up-regulated	1	0.002	**3.583**	0.001	**2.982**
	*Hpse*	Up-regulated	1	**2.174**	1.227	1.17	**3.223**
	*Itga11*	Up-regulated	1	**20.773**	**40.039**	**100.962**	**316.34**
	*Itga5*	Up-regulated	1	1.986	**3.011**	**2.995**	n.d.
	*Itgb8*	Up-regulated	1	1.538	**2.21**	**4.789**	**6.166**
	*Klra2*	Up-regulated	1	1.73	1.279	**4.221**	**11.168**
	*Mcam*	Up-regulated	1	0.967	1.687	1.894	**2.126**
	*Pigc*	Up-regulated	1	1.08	1.521	1.714	**2.475**
	*Renbp*	Up-regulated	1	1.926	**2.156**	**2.155**	**2.21**
	*Fuk*	Down-regulated	1	**0.428**	0.636	0.559	0.573
	*Pmm1*	Down-regulated	1	**0.499**	0.857	1.042	1.013

n.d.: “not determined”.

**A.** Group of genes with the same variation during myogenic differentiation of C2C12 and satellite cells. **B.** Group of genes with variation during myogenic differentiation of satellite cells and no variation during C2C12 differentiation. Numbers in bold were considered as significant variations represented by relative mRNA quantities less than 0.5 or up to 2. n.d.: not determined.

### “Without *a priori”* clustering analysis

We performed clustering analysis in order to group genes with similar expression patterns. An unsupervised hierarchical clustering algorithm was used to study 67 genes. This approach, described in Materials and Methods, relies on the time course comparison of differential expression of each gene when C2C12 and MSC strains are analyzed.

**Figure 4:**
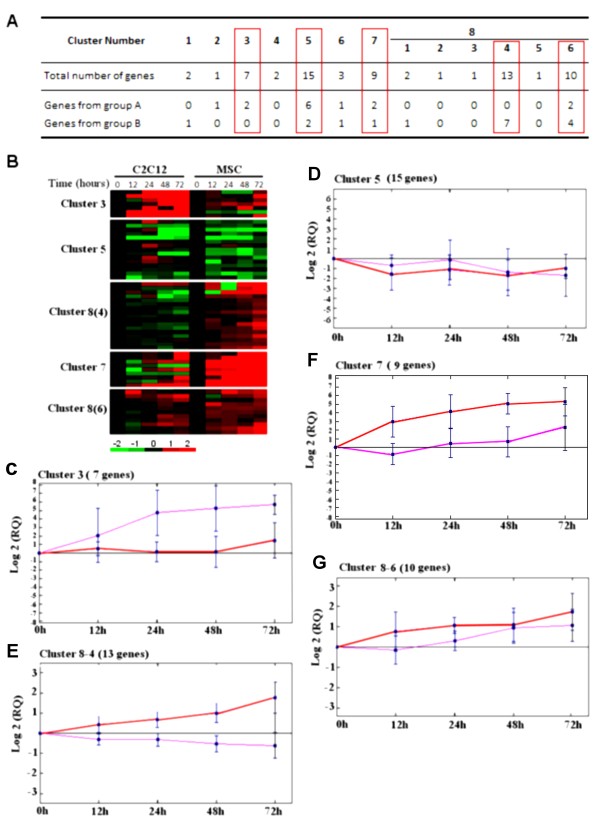
**Clustering analysis of variant genes during myogenesis.** Expression profiles of the 67 genes whose transcript levels changed significantly during myogenesis of mouse satellite cells were used. The resulting tree of hierarchical clustering (see Methods) was split into eight groups (1 to 8). Group 8 included 28 genes and was split again into six smaller groups (1 to 6) to obtain more homogenous groups. **A.** Number of genes in clusters and number of genes found in group A and group B as defined in Table 1. Mean expression profiles for C2C12 (pink) and MSC (red) of clusters 3, 5, 7 8(4) and 8(6) are shown in **C**, **D**, **E**, **F** and **G**. **B.** Expression map of the five major clusters (boxed in red): 3, 5, 7, 8(4) and 8(6). **C-G.** Mean expression profiles obtained for C2C12 (pink) and MSC (red): *y*-axis represents the standard log2 of expression levels (RQ for relative quantity). Error bars indicate standard deviation of average expression.

The resulting tree was split into eight groups (some details of the subdivisions over the last group (group 8) are given (6 sub-clusters can be identified in this group)) (Figure 4A). 26 genes (from group A or B) fall into the 5 main groups (3, 5, 7, 8(4), 8(6)) (including at least seven genes). 12 of the fourteen genes of group A are split into clusters 3, 5, 7 and 8(6) (Figure 4A). In cluster 3, gene expression increased early in differentiation (a clear up-regulation) for C2C12 and only at 48 hours for MSC (Figure 4C). Cluster 5 contains 6 genes from group A with similar expression profiles in both conditions: these genes were clearly down-regulated (Figure 4D). In cluster 8(6), all genes were up-regulated and their expression variation was higher in MSC than in C2C12 (Figure 4G). This cluster also includes 4 genes from group B (Figure 4B). Group 8(4) also contains 7 genes from group B which had a significant variation in expression only in MSC (Figure 4E). The remaining group B genes are split between clusters 5 (2 genes), 7 (1 gene, Figure 4F) and 3 minor groups (1, 6 and 8(1)). We observed that 26 of the 31 genes selected were gathered “without *a priori*”. It demonstrates the confidence we can have in our selection method.

### Myogenesis requires the presence of adhesion proteins and the sulfation of keratans

As shown in Table 1, we observed the highest changes in expression for *Chst5, Itga11* genes which were the most up-regulated (54 and 316 fold, respectively) and *Itgb7* which was down-regulated (0.16 fold). To understand what cell processes could be affected by these expression variations, we classified the 31 genes according to their function [23-51] (Table 2). We showed that a large part of these genes encoded adhesion proteins (around 30%), and only one of them was down-regulated. For the remaining 19 genes, 4 genes are involved in sulfation with Chst5 which specifically sulfates the keratans. The 7 down-regulated genes were unrelated to myogenesis (e.g.: the *Clgn* product is involved in spermatogenesis [48]).We observed a difference with the study of Janot *et al.*[15] for genes involved in lacto/neolacto series biosynthesis. Indeed, we found none of the seven genes previously described. They were discarded because they varied in both myogenic and early adipogenic pathways.

**Table 2:** Classification of the 31 selected genes

**Gene symbol**	**Expression pattern**	**Function**	**Family**
*Art1*	Up-regulated	ADP-ribosylation	**Adhesion familly**
*Cd248*	Up-regulated	Potential angiogenesis role	
*Clec2d*	Up-regulated	Osteogenesis inhibitor	
*Icam2*	Up-regulated	Cell-cell interaction	
*Itga5*	Up-regulated	Matrix remodeling	
*Itga6*	Up-regulated	Laminin receptor	
*Itga11*	Up-regulated	Collagen receptor	
*Itgb8*	Up-regulated	Fibronectin receptor	
*Klra2*	Up-regulated	Myosin heavy chain receptor	
*Lgals7*	Up-regulated	Cell-cell and cell-EMC interaction	
*Mcam*	Up-regulated	Cellular adhesion	
*Itgb7*	Down-regulated	Lymphocyte homing and retention	
*B4galt1*	Up-regulated	Keratan sulfate biosynthesis	**Glycanic synthesis/extension**
*Chst12*	Up-regulated	Dermatan sulfate biosynthesis	
*Cmah*	Up-regulated	CMP-N-acetylneuraminic acid hydroxylase	
*Chst5*	Up-regulated	Keratan sulfate biosynthesis	
*Galntl1*	Up-regulated	O-Glycan core biosynthesis	
*Gcnt2*	Up-regulated	O-Glycan core biosynthesis	
*Has1*	Up-regulated	Hyaluranane synthase	
*Has2*	Up-regulated	Hyaluranane synthase	
*Hpse*	Up-regulated	Heparanase	
*Renbp*	Up-regulated	Epimerase	
*Pigc*	Up-regulated	GPI anchor biosynthesis	
*Chst8*	Down-regulated	N-Glycan sulfation	
*Chst10*	Down-regulated	O-Glycan sulfation	
*Pmm1*	Down-regulated	Phospho-manno mutase	
*Clec4d*	Down-regulated	Endocytic receptor	**Other functions**
*Clgn*	Down-regulated	Potential spermatogenesis role	
*Fcna*	Up-regulated	Ficollin	
*Fut10*	Down-regulated	Chitobiose fucosylation	
*Fuk*	Down-regulated	Fucose recycling	

The selected 31 genes (Table 1) were classified according to the function of their products.

### Decreased expression of *Itga11* inhibits cell fusion

**Figure 5:**
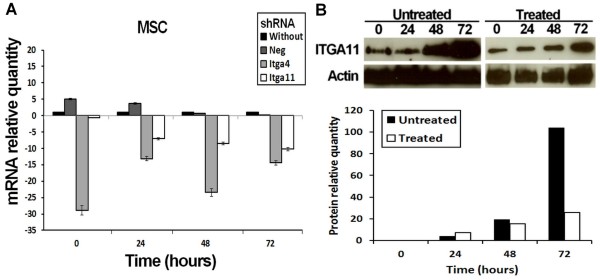
***Itga11 *knockdown decreased mRNA and protein amount. A.** Relative quantities of *Itga4* (soft grey) and *Itga11* (white) mRNA for treated cultures, or untreated cultures (black) in MSC. For treated cultures with negative control shRNA (dark grey), both *Itga11* and *Itga4* mRNA were quantified but to simplify, only the relative quantity of *Itga11* is shown. **B.** Detection of ITGA11 by western blot during myogenesis. Proteins were extracted from MSC culture, before (0 h) and during differentiation (24 h, 48 h and 72 h) and the quantities of ITGA11 were revealed by western blot using Actin as control.

**Figure 6:**
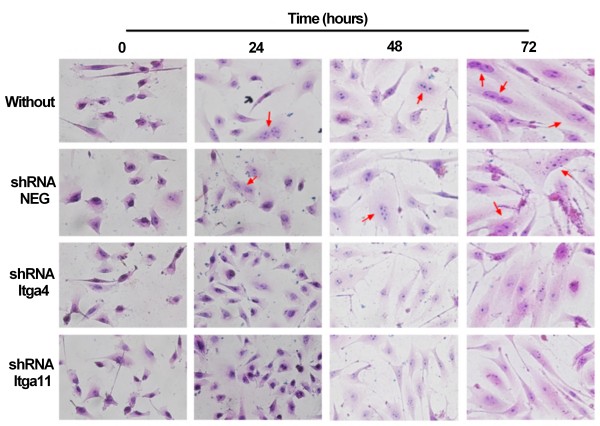
***Itga11 *knockdown reduced myotube formation.** Myogenic differentiation of MSC: untreated (Without), treated with plasmid containing shRNA without target in murine genome (shRNA NEG), treated with plasmid containing shRNA for *Itga4* (shRNA *Itga4*) or plasmid containing shRNA for *Itga11* (shRNA *Itga11*). Photos were taken at different time points after hematoxylin/eosin staining. Magnification was 400× (for lower magnification see Additional file 3). Red arrows point myotubes.

**Figure 7:**
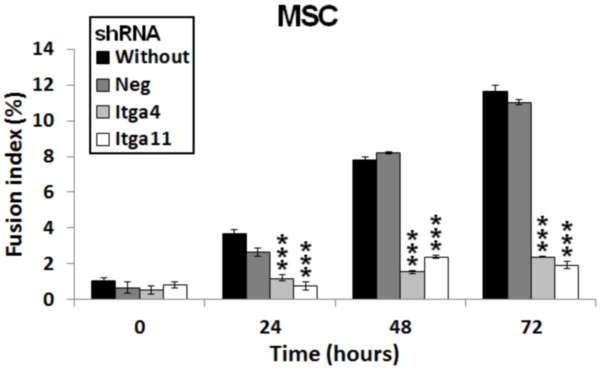
***Itga11 *knockdown decreased fusion index.** Fusion index of MSC cultures seeded at 5×10^3^ cells/cm^2^ on Matrigel®. Cultures were compared every 24 h after myogenic differentiation induction by serum starvation. Cultures were untreated (black), treated with plasmid containing negative control shRNA (dark grey), treated with plasmid containing shRNA for *Itga4* (light grey) or for *Itga11* (white), see Methods. Standard errors were calculated using triplicate and the Student t-test determined significant differences between control and treated cultures. ***p < 0.1%.

We observed an increase of ITGA11 in MSC during myogenic differentiation (Figure 5). These results correlated well to previously observed mRNA levels (Table 1). Gene repression using shRNA against *Itga11* and *Itga4* (as a control due to its well-known implication in fusion [52]) was performed on MSC 24 hours before induction of myogenic differentiation and repression was verified by RT-PCR (Figure 5A). The amount of ITGA11 was dramatically reduced in treated MSC although it slightly increased during differentiation (Figure 5B). A negative plasmid containing shRNA without any target in the murine genome was used as control. It showed an up-regulation of *Itga11* before differentiation (Figure 5A) that did not affect the fusion ability of the cells (Figure 6). shRNA transfected cells (against *Itga11* or *Itga4*) showed a large decrease mRNA level in MSC (Figure 5A). This resulted in a low number of myotubes in treated culture after 72 hours of myogenic differentiation (Figure 6). The fusion index obtained for untreated MSC cells or cells treated with the negative control shRNA did not display a significant difference (Figure 7). A significant inhibition of MSC fusion occurred and the fusion index only reached 2.5% for shRNA treatment after 72 hour differentiation (Figure 7).

### Itga11 antibodies inhibited the cell fusion

**Figure 8:**
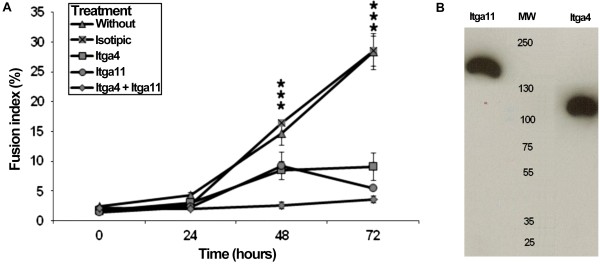
**Anti-ITGA4 and/or anti-ITGA11 antibodies inhibited cell fusion of myoblasts. A.** Experiments were performed on cultures plated on Matrigel® and under differentiation conditions. The five cultures were, untreated (triangles), treated with 1 μg/ml isotypic antibodies as negative control (cross over squares), treated with 1 μg/ml anti-ITGA4 antibodies as positive control (squares), treated with 1 μg/ml anti-ITGA11 antibodies (circles) or treated with 0.5 μg/ml anti-ITGA4 and 0.5 μg/ml anti-ITGA11 antibodies (diamonds). Significance was calculated using the t-test on three simultaneous replicates (see standard bars). ***indicates time with significant difference between WT and all neutralization tests, p <0.1%. **B.** The specificity of anti-ITGA4 and anti-ITGA11 was controlled by western blot using 50 μg of cellular lysate. MW indicates the molecular weight ladder.

Regarding the effect of *Itga11* down-regulation, we proceeded to study the neutralization of ITGA11 during differentiation. Treatments were performed on MSC cultures with antibodies against ITGA11 or ITGA4 (as control). After 3 days, the fusion index was significantly reduced for both treatments (Figure 8). At 72 hours, the fusion indexes of treated cells were reduced by approximately 65% in both cases. In MSC cultures containing both antibodies, the fusion index was decreased by 90% after 72 hour differentiation (Figure 8). This result indicated a direct involvement of ITGA11 in the MSC fusion and 2 different effects of both integrins in this process.

### Decreased expression of *Chst5* delayed the cell fusion

**Figure 9:**
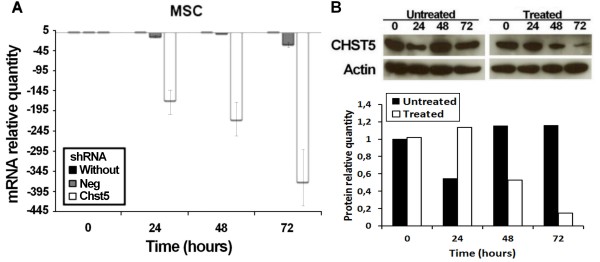
***Chst5 *knock-down decreased mRNA and protein amount. A.** Relative quantities of *Chst5* mRNA for treated cultures (white), or untreated cultures (black) in MSC and for treated cultures with negative control shRNA (dark grey), *Chst5* mRNA were quantified by RT-PCR. **B.** Western blot detection of CHST5 during myogenesis. Proteins were extracted from MSC culture, before (0 h) and during differentiation (24 h, 48 h and 72 h) and the relative quantities of Chst5 were revealed by western blot using Actin as control.

**Figure 10:**
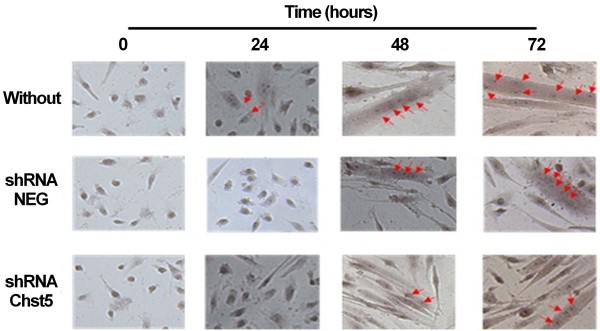
***Chst5 *knockdown induced a late in myotube formation.** Myogenic differentiation of MSC: untreated (Without), treated with plasmid containing shRNA without target in murine genome (shRNA NEG), treated with plasmid containing shRNA for *Chst5* (shRNA Chst5). Photos were taken at different time points after hematoxylin/eosin staining. Magnification was 400x (for lower magnification see Additional file 3). Red arrows point nucleus into myotubes.

**Figure 11:**
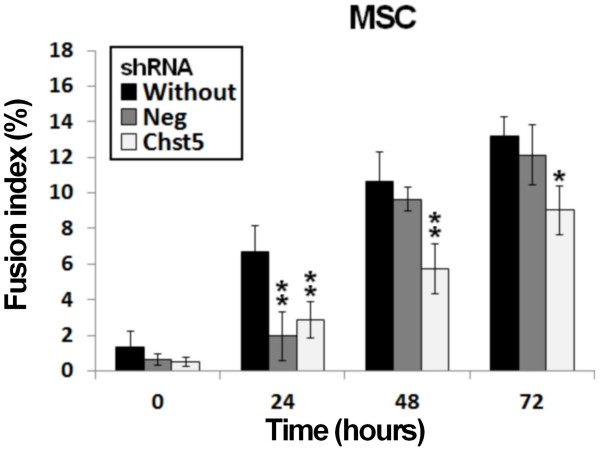
***Chst5 *knockdown delayed the cell fusion.** Fusion index of MSC cultures seeded at 5×10^3^ cells/cm^2^ on Matrigel®. Cultures were compared every 24 h after myogenic differentiation induction by serum starvation. Cultures were untreated (black), treated with plasmid contains negative control shRNA (dark grey), treated with plasmid containing shRNA for *Chst5* (white), see Methods. Standard errors were calculated using triplicate and the Student t-test determined significant differences between control and treated cultures. **p < 1%; *p < 5%.

We observed a large up-regulation of *Chst5* during the differentiation of MSC. So we treated MSC cultures with shRNA against *Chst5* and controlled the induced repression by RT-PCR (Figure 9A). We also controlled the correlation with the protein amount by western blot in MSC (Figure 9B). The treated cultures showed a decrease of *Chst5* expression and a lower protein amount. After 24 hours in the differentiation condition, the fusion index observed in culture treated with shRNA against *Chst5* was significantly lower than that observed for the untreated culture (Figures 10 and 11). This difference remains until 72 hours of differentiation but appeared to decrease. This indicates that *Chst5* inhibition only induced a delay in MSC fusion. This result and those obtained for *Itga11* strengthen our gene selection.

## Discussion

Cell migration, adhesion and fusion require many changes in the cell surface and environment during myogenesis. Janot *et al.* (2009) used a RT-PCR-based screening method to detect 37 glycosylation related genes (GRG) with a large variation in expression during the early myogenic differentiation of C2C12 [15]. However, the genes specifically involved in myogenesis were not distinguished from those that were expressed independently from a differentiation pathway and those which were associated with C2C12 immortality. In this study we refined the screening using murine satellite cells (MSC) since (i) they better reflected the *in vivo* state and (ii) they can be differentiated into myotubes or into early fat storage cells. By comparing 383 GRG (Additional file 4) expression in MSC differentiated in one or the other differentiation pathway we retained 67 genes specifically associated with the myogenesis out of 383 studied.

Compared to GRG previously found by Janot *et al.,* these 67 genes can be divided into two groups. The first one contains 17 genes whose expression varies in only MSC. The second is composed of 50 genes with variation in both C2C12 and MSC, 14 only showing the same pattern of expression. We did not retain the 36 other genes because differences in their expression profiles seemed to depend on cell type rather than on myogenesis. The discrepancy between these results and the ones previously published by Janot and co-workers is mainly due to a more drastic screening that discards genes common to pre-adipogenic and myogenic differentiation. So, we retained 31 genes that seem specifically involved in myogenic differentiation.

We strengthened our selection by using “without *a priori*” clustering of GRG selected in the MSC model. We found 26 of the previously selected 31 genes were distributed into 5 clusters among the 13 clusters obtained. Cluster 5 included all 8 down-regulated genes. 6 of them had a similar variation during C2C12 and MSC myogenesis (Table 1, group A). Since the other 23 genes were all up-regulated in C2C12 and/or MSC, they were mainly found in 4 clusters: clusters 3 and 7 which contain 4 group A genes and 1 group B gene; clusters 8(4) and 8(6) which contain 11 group B genes and 2 group A genes. This demonstrates that our separation and the distribution by “without *a priori*” clustering of the GRG were very close.

To explore the relationship between GRG up- and down-regulation, and myogenesis, we classified them according to the function of their products (Table 2). Our first observation was for Art1, one of the most up-regulated genes, it encodes a mono-ADP-ribosyltranferase with a specific expression in myotubes. 14 of the classified genes encoded proteins, which are involved in glycan synthesis or modifications such as sulfation (e.g.: *Chst5*) or hydrolysis (e.g.: *Hpse*). Most of them are responsible for extracellular matrix synthesis (*B4Galt1*, *Chst5*, *Chst12, Cmah*, *Galntl1*, *Gcnt2*, *Has1* and *Has2*); they are up-regulated while those coding for sulfation of glycans carried by glycolipids or glycoproteins are down-regulated (*Chst8* and *Chst10*). The alignment of cells and myotubes requires a different structural organization of the extracellular matrix molecules such as lumican, composed of keratan sulfates [53]. In this study, an up-regulation of *B4Galt1* and *Chst5* involved in keratan sulfate biosynthesis was observed. The latter also depends on the synthesis of *O*-glycan core 2 structure. 2 up-regulated genes encoding proteins of the *O*-glycan biosynthesis pathway, *Galntl1* and *Gcnt2,* are also implicated. In addition, considering all up- or down-regulated GRG during myogenic differentiation, without taking into account their variation in early adipogenesis, we found that GRG expression variations were more favourable for core 2 biosynthesis (Additional file 5). Indeed, *Gcnt1* was up-regulated in both differentiation pathways. However, in myogenic cells, biosynthesis continued to keratan sulfate since *B4Galt1* and *Chst5* gene expression increased. So, we propose that the regulation of some GRG contributes to core 2 *O*-glycan biosynthesis and subsequently that of keratan sulfates and lumican for myotube arrangements. Finally, it has been shown recently that Gcnt1 up-regulation is associated with myocardial hypertrophy in mice [54], which supports our theory.

Significant adhesion protein involvement during cell fusion is likely since 12 of the 31 genes encode proteins of this family. Among them, we observed an up-regulation of a protein containing a lectin domain, CLEC2D, a murine osteoclast inhibitory lectin [25] necessary to promote MSC myogenesis. The LGALS7 lectin plays a key role in stabilization of glycoconjugates in epithelial repair [31]. It could play a similar stabilizing role in myogenesis. The up-regulation found for CD248 or Endosialin, known as a potential actor of angiogenesis in which it contributes to cell-cell alignment and contacts [24], seems to be explained. Again, up-regulation of KLRA2 (or Ly-49), a cell surface receptor of class 1 myosin heavy chain, could be related to the high expression of its ligand in skeletal muscle [30].

**Figure 12:**
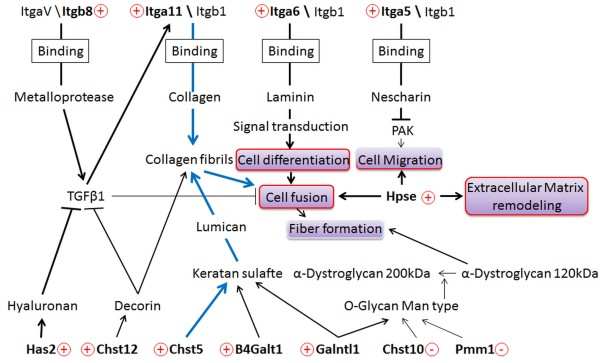
**Interaction network leading to cell fusion during myogenesis.** The network was constructed according to the literature and based on twelve variant genes. Genes are in bold (+: up-regulated or -: down-regulated). The thickness of the lines is proportional to the expression levels of genes during myogenesis. Blue arrows show the pathways checked by functional experiments. Myogenic steps are in purple rectangles and boxed in red when cells are engaged in this step and in white for later steps.

We also found into adhesion proteins 5 integrin subunits (ITGA5, ITGA6, ITGA11, ITGB7 and ITGB8) and 2 adhesion molecules (ICAM2 and MCAM). ICAM2, whose expression increases during C2C12 and MSC myogenesis, is notably expressed in adult satellite cells [55]. It can link α-actin/actin to form a membrane-actin interaction that reinforces cell-cell interactions as described in neuroblastomas [26]. MCAM is known to have a role in cellular adhesion and in cohesion of the endothelial monolayer [32]; it could have the same role in myogenesis. Moreover, Cerletti *et al.* reported that *Mcam* gene is highly expressed in human foetal myogenic cells [56]. Therefore, there is a good relationship between this result and the up-regulation found in MSC. The integrin family is of particular interest and is often involved in cell contact, signaling, adhesion and fusion [19]. From our results, a network model (Figure 12), with a central role for the integrins, is proposed to partially explain myogenesis regulation. The *Itga5* gene encoding an integrin subunit (ITGA5) was up-regulated. ITGA5 forms a heterodimeric complex with ITGB1 and then with Nischarin during control of cell migration [57]. Interestingly we observed that *Hpse* was also up-regulated. The protein encoded by this gene (Heparanase) is known for its role in cell migration through the digestion of ECM, creating a balance with ITGA5 activity [41]. We noticed the up-regulation of *Itgb8*. The ITGB8 protein, associated with ITGAV and a metalloprotease, activates transforming growth factor β-1 (TGF-β1) in epithelial cells or neurons [27,28]. Although TGF-β1 is known to inhibit myogenic differentiation [58], Gouttenoire *et al*. demonstrated that it can also activate *Itga11* transcription in mouse chondrocytes [59]. In addition, genes encoding hyaluronan synthases HAS1 and HAS2 are up-regulated and hyaluronans are described to promote sequestration of the TGF-β1 receptor in lipid rafts which limits their interaction with TGF-β1 [60]. Finally, up-regulation of *Chst12* involved in dermatan sulfate biosynthesis may indicate an increase in the production of decorin, known to bind and regulate the sensitivity of chicken satellite cells to TGF-β1 [61]. All results suggest that cells could regulate TGF-β1 effects through different pathways and may limit its action to *Itga11* activation (described later). Our model also used cell signalling according to the up-regulated integrin subunit ITGA6. It forms a complex with ITGB1which has recently been described as essential for neuronal differentiation of human embryonic stem cells. The binding of the complex to laminin molecules induced differentiation [29] so we integrated a similar system in our model of myogenesis. Recently, ITGA6 was linked to the expression of cell migration-related genes [62], it could be also play a role in cell migration as ITGA5.

Laminin can also bind α-Dystroglycan and organize muscle structure. The 120 kDa form of α-Dystroglycan, in chicken myoblast cultures, is present at the late stage of myogenesis [63]. This form is likely to be less rich in mannose type *O*-glycan. In our model, we associated the decrease in *Pmm1* and *Chst10* expression with this phenomenon. Because PMM1 is involved in UDP-mannose synthesis and the sulfotransferase, CHST10, is responsible for terminal sulfation of *O*-mannosyl glycans, they are presumably involved in cell adhesion [64] and could be related to the structures present on the α-Dystroglycan molecule [65].

Earlier, we showed that *Itga11* showed the highest variation in expression. This gene encoding the ITGA11 subunit has also been shown to be up-regulated during myofibroblast differentiation in cardiomyopathy [66]. We included this integrin in our model because it has already been described to be produced by human corneal myoblasts and to be involved in development of the latter [67]. To verify the involvement of selected GRG in myogenesis, *Itga11* and *Chst5* were chosen for a functional study. Treatment of MSC with shRNA directed against the *Itga11* transcript before and during myogenic differentiation strongly inhibited cell fusion. ITGA11 depletion stopped the fusion after 24 hours only (Figure 8). The MRFs expressions were followed during the differentiation of treated cells and we observed a complete inhibition of the Myf6 expression (Additional file 6A). The expression of Myf6 is usually related to the fusion process. The addition of anti-ITGA11 antibodies strongly inhibited fusion; after 72 hours, under differentiation conditions, less than 7% fusion was observed. The same result was obtained with anti-ITGA4 antibodies and ITGA4 is known for its involvement in fusion during myogenesis [52]. The combined action of antibodies against both integrins completely inhibited cell fusion. This result demonstrated that the effect of each antibody could be cumulative (or synergistic). Since no compensations were observed in shRNA experiments, we suggest that the two integrins contribute independently to the binding of cells to different MEC components. Now it is clear that ITGA11 plays a role during myogenesis, especially in the fusion process. The cells treated with shRNA against *Chst5* showed a delay in fusion during the first 48 hours of differentiation. However, they recovered a fusion index quietly similar to untreated cells at 72 hours under differentiation conditions. This effect could be explained by the involvement of CHST5 in the sulfation of keratans. The low amount of CHST5 during 48 hours of differentiation was not sufficient to permit enough sulfation of keratans and thus the cell fusion. Beyond of the 48 hours, the keratan sulfate amount was sufficient to allow a quick fusion of cells. Indeed, at 72 hours of differentiation the fusion index was close between treated and untreated cultures. This result correlated to the MRFs expression observed during the differentiation of treated cells (Additional file 6B). Indeed, MyoG up-regulation during the first 24 hours showed that cells are engaged in myogenesis. The Myf6 expression increased only from 48 hours and harshly rose until 72 hours as observed for the fusion index. These results demonstrate the importance of *Chst5* to initiate the fusion step of myogenesis. The relationship between *Itga11, Chst5* and myogenesis step strengthens our selection as well as our myogenic regulation model.

## Conclusion

In this study, the comparison between the adipogenic and myogenic differentiation of MSCs, as well as between two cell types (C2C12 and MSC) that differentiate into myotubes allowed us to select 31 genes whose expression is particularly associated with myogenesis. We classified these genes into 3 groups, according to the function of the proteins they encode: (i) remodelling of the extracellular matrix where genes are predominantly up-regulated such as *Chst5*; (ii) glycan biosynthesis with more particularly an up-regulation of genes involved in core 2 glycan synthesis; (iii) adhesion where genes are mainly up-regulated such as *Itga11*. We have also shown that *Itga11* knockdown and neutralization of ITGA11 protein strongly inhibit cell fusion whereas the knockdown of *Chst5* delayed the fusion of myoblast. These results emphasize our selection of genes and their involvement in myogenesis. We suggest a potential regulation network for myogenesis in which some GRGs are strongly implicated. Finally, this study provides a suitable complementary method for the study of specific differentiation pathways.

## Methods

### Cell cultures

The murine C2C12 myoblast cell line (ATCC, Manassas, VA, USA) was cultured in DMEM (Dulbecco’s modified Eagle’s medium, Eurobio, Courtaboeuf, France) supplemented with L-Glutamin, 10% (v/v) fetal calf serum (Eurobio), 50 units/mL penicillin and 50 μg/ml streptomycin. Cells were grown to 80% confluence and differentiated into myotubes in DMEM supplemented with 5% (v/v) horse serum (Invitrogen, Carlsbad, CA, USA). The medium was changed every 48 hours.

Murine satellite cells were extracted from posterior leg muscles of C3H mice as previously described [68]. Cells were cultured in “medium A” containing HAM F10 medium (Sigma) supplemented with 5 mM L-glutamin, 20% (v/v) horse serum, 50 units/mL penicillin, 50 μg/mL streptomycin and 5 ng/mL Basic-Fibroblast Growth Factor (Sigma). At 70% confluence cells were differentiated into myotubes with HAM F10 medium supplemented with 10% (v/v) horse serum or trans-differentiated into fat storage cells when 50 mM glucose were added to “medium A”.

### Determination of cell fusion index and staining of early fat storage cells

After removing culture medium, cells were washed twice with PBS, fixed with 10% (v/v) formalin for 15 min at room temperature and washed twice again with PBS. Nuclei were stained with Shandon Harris hematoxylin (0.44% (v/v), Thermoscientific, Courtaboeuf, France) for 1 hour at room temperature and then washed twice with PBS. Cytoplasm was stained with Shandon eosin Y aqueous (0.5% (v/v), Thermoscientific) for 30 min at room temperature. Fusion index of C2C12 and satellite cells was determined by the ratio between nuclei in myotubes and total nuclei on six different microscopic fields.

To identify early fat storage cells, fixed cells (10% (v/v) formalin, 30 min, 37°C) were stained with 0.3% Oil-Red-O (Sigma) in 60% isopropanol according to the protocol described by Salehzada *et al.*[69].

### Quantitative real-time PCR (QRT-PCR)

For each kinetic point, cells were rinsed twice with PBS and harvested following trypsinization (PBS, 1 mM EDTA, 0.05% (w/v) trypsin). Total RNA was extracted using the RNeasy mini Kit (Qiagen Inc., Hilden, Germany). A micro-fluidic chip was used to measure quality and quantity of total RNA (Agilent 2100 Bioanalyser, Agilent Technologies Inc., Santa Clara, CA, USA) and 1 μg was converted into cDNA using the High Capacity cDNA Archive Kit (Applied Biosystems, Foster city, CA, USA).

mRNA was quantified by QRT-PCR on ABI Prism 7900 Sequence Detector System using TaqMan probe-based chemistry (Applied Biosystems), with 6-carboxyfluorescein (FAM) as a reporter. cDNA (2 ng) was used to quantify myogenic and adipogenic markers and target genes in 96-well plates and Taqman Low Density Array (TLDA) respectively. In addition to genes already present on TLDA as previously described by Janot *et al.*[15], we also followed the expression of 8 genes encoding integrin subunits (*Itga1*, *Itga8*, *Itga10*, *ItgaD*, *ItgaE*, *ItgaV*, *Itgb1*, and *Itgb6*) to complete this gene family. Relative quantification was performed using five reference genes: *18S RNA, G6pdx, Gapdh, Tcea, Tbp*.

### Data analysis and clustering

mRNA gene transcription data were collected and analyzed using SDS 2.2.2 software (Applied Biosystems). The first accessible data was the Ct, the minimum number of cycles necessary to obtain a significant fluorescent signal; therefore genes with a Ct above 35 were considered as not expressed. Relative quantification was obtained using the ∆∆Ct method [15]. mRNA quantity was normalized using Cts from *Gapdh*, *Tbp* and *18S RNA* and t = 0 h was used as a reference sample. The evolution of the expression level along the kinetic points was also considered to discard point showed aberrant relative quantity (*e.g.* RQ = 0.001 or RQ = 10000). The relative quantity for these points was indicated as “not determined” (n.d.).

The following process for distance computation as well as hierarchical clustering was performed with a script, personally communicated by G. Lelandais and previously used by Lucau-Danila *et al.*[70]. All genes for which a change of more than two folds was observed at least once during the experiments were selected using Log2 (ratio) data sets of time courses. A cluster analysis was conducted on similarity measurements between the differential gene expression profiles. More precisely, each gene can be given a coordinate of two expression vectors defined as C2C12*g*(*Tm*) and SatC*g*(*Tm*), where C2C12*g*(*Tm*) and SatC*g*(*Tm*) are the logarithms to base 2 of the ratios for gene *g* in C2C12 or satellite cells respectively, measured at *Tm*, the time point of the kinetic [*Tm* which is included in *T* (12 h, 24 h, 48 h, 72 h)]. Thus, considering two genes, *g*1 and *g*2, the similarity (D) between them is computed as follows:

$$D\left(g_{1}g_{2}\right)\;=\;\sum_{T_{m}\in T}\sqrt{{\left[C2C12_{g1}\left(T_{m}\right)\;‒\;C2C12_{g2}\left(T_{m}\right)\right]}^{2}\;+\;{\left[\mathrm{Sat}C_{g1}\left(T_{m}\right)\;-\;\mathrm{Sat}C_{g2}\left(T_{m}\right)\right]}^{2}}$$

A complete-linkage hierarchical clustering analysis was performed next, by using the distance matrix. Initially, each object is assigned to its own cluster and then, at each stage, the two most similar clusters are joined by the algorithms that proceed iteratively. The process continues until the analysis reaches a single cluster. The resulting tree is finally split into several groups of genes. Library function in R was used [http://cran.r-project.org], and graphical representations were obtained with MeV (MultiExperiment Viewer v 4.7.4) [71].

### Immuno-neutralization experiments

One day before induction of differentiation, satellite cells were treated with 1 μg/mL isotypic antibodies (purified sheep IgG, R&D systems), rat anti-ITGA4 or sheep anti-ITGA11 (R&D systems, Minneapolis, MN, USA). Treatments with anti-ITGA4 or isotypic antibodies were used as positive or negative controls respectively. Immuno-neutralization was maintained by addition of antibodies every 24 hours at the same concentration. For these kinetic points, the fusion percentage was determined using culture without antibody as assay.

### Knockdown by ShRNA

MSC were seeded at 5×10^3^ cells/cm^2^ on Matrigel®-coated plates, cultured in growth medium for 2 days and cultured in differentiation medium for 3 days. The medium was changed every 24 hours and cells were treated during the days 2 and 3. Treatments were 200 μL medium containing 4 μg plasmid and 2 μL transfecting reagent (Attracten, Qiagen). Cells were untreated, treated with a plasmid containing shRNA without target, treated with plasmid containing shRNA targeting *Itga4* or treated with plasmid containing shRNA targeting *Itga11* or *Chst5*. Cultures were stopped and stained every 24 hours during differentiation and the fusion percentage was calculated. Total RNA was also collected and knock-down of *Itga4*, *Itga11* and *Chst5* expression was verified by PCR with *Gapdh* as control. Probes used were: *Itga4*-Forward: AGACCTGCGAACAGCTCCAG; *Itga4*-Reverse: GGCCTTGTCCTTAGCAACAC; *Itga11*-Forward: GGCCGCCTTCCTCTGCTTCA; *Itga11*-Reverse: TTGCCACCCCTGGTGGCGAT; *Chst5*-Forward: CTGAGCGGCTCTTTGTGTGC and *Chst5*-Reverse: TCAAGGAGGTGCGCTTCTTT. The relative quantity was determined as follows: (i) the area and mean grey values were taken into account, (ii) obtained values were normalized to *Gapdh* (iii) normalized values for the control culture were assigned a value of 1 for each time point (iv) final ratios were equal to normalized values obtained for treated cultures at a certain time points divided by values for untreated cultures at the same times.

### Western blot analysis

At each time point of differentiation, cell proteins were extracted with Triton buffer (Tris 50 mM Tris, 0.5% (v/v) Triton X-100, 0.5% (w/v) sodium deoxycholate, pH7.4, protease inhibitor cocktail (Roche, Boulogne-Billancourt, France)). Proteins (50 or 100 μg protein loaded per lane) were resolved by SDS-PAGE using 10%-polyacrylamide gels. Proteins were transferred to nitrocellulose membrane for 90 minutes at 0.8 mA/cm^2^. Membranes were saturated with TBS (20 mM Tris,137 mM NaCl, pH7.6) supplemented with 0.1% (v/v) Tween 20 and 2.5% (w/v) powdered skim milk, for 1 h 30 at room temperature. Blots were probed with anti-ITGA11 (1 μg/mL at 4°C over-night, R&D systems) or anti-CHST5 antibodies (1 μg/mL at 4°C over-night, Bios) followed by peroxidase coupled goat anti-rat IgG for ITGA11 (1:1000 for 1 h 30 min at room temperature, R&D systems) or by peroxidase coupled swine anti-rabbit IgG for CHST5 (1:1000 for 1 h 30 min at room temperature, Dako). Bands were visualized by enhanced chemoluminescence (CN 11500694001, Roche). Membranes were washed 3 times with TBS-0.05% (v/v) Tween after incubations.

### Availability of supporting data

The data set supporting the results of this article is included within the article (Additional file 7).

## Competing interests

The authors declare that they have no competing interests.

## Authors’ contributions

VG contributed to the conception, acquisition end analysis of all data and drafted the manuscript. ADS performed the statistical analysis by clustering. JS contribute to the experiments related to *Chst5*. AM has been involved in manuscript drafting. FD provided the previous data on C2C12and helped to formulate new experiments on these cells. JMP supervised all the work, conceived the study, participated in its design and coordination, and helped to draft the manuscript. All authors have read and approved the final manuscript.

## Supplementary Material

Additional file 1 Satellite cells differentiate into myotubes or early fat storage cells. A, B. Satellite cells at t = 72 h under myogenic differentiation conditions, plated on Matrigel®, without (A) or with (B) hematoxylin/eosin staining. Magnification 100×. Arrows point myotubes. C, D. Satellite cells under early adipogenesis trans-differentiation conditions, plated on Matrigel®, at differentiation time t = 168 h. (C) before and (D) after staining with Oil-Red S. The black arrows show lipid accumulation in cells and red arrow shows the nucleus. Magnification 400×. Click here for file

Additional file 2 Sixty-seven genes regulated only during myogenic differentiation of MSC. List of up or down regulated genes during myogenic but not adipogenic MSC differentiation and their expression variation. Click here for file

Additional file 3 shRNA against *Itga11* and *Chst5* inhibit and delayed fusion respectively. Myogenic differentiation of MSC: untreated (Without), treated with plasmid containing shRNA for *Itga11* (shRNA Itga11) or shRNA for *Chst5* (shRNA Chst5). Photos were taken at different time points after hematoxylin/eosin staining. Magnification was 100×. Click here for file

Additional file 4 Three hundred and eighty three genes under study. List of the 383 genes selected from the murine glyco-genome and used for our screening. Click here for file

Additional file 5 Orientation of O-glycan biosynthesis. Mucine type O-glycan biosynthetic pathway representation with its enzymes (+: up-regulated, - : down-regulated, H: high constant expression, L: low constant expression) during myogenic differentiation. Red lines symbolize the activated synthetic pathways and the black lines the repressed ones. Core and F1α correspond to the name of the glycan structures: (●) all genes leading to this structure are expressed; (○) some genes in the pathways have no or very low expression. Modified from KEGG Pathway (http://www.genome.jp/kegg/pathway.html). Click here for file

Additional file 6 MRFs expression during differentiation of shRNA-treated cells. A.B. Expression of the MRFs (*MyoG* (circles), *MyoD1* (squares), *Myf5* (diamonds), *Myf6* (triangles)) during the differentiation of satellite cells treated with shRNA against *Itga11* (A) or *Chst5* (B). Click here for file

Additional file 7 Data supporting this article. Excel table including all TLDA results for C2C12 and satellite cells experiments. Click here for file
