# Supplementary figures and images for: Highlights of glycosylation and adhesion related genes involved in myogenesis
Source: BMC Genomics. 2014 Jul 22;15:621. doi: 10.1186/1471-2164-15-621 (PMC4223822; doi:10.1186/1471-2164-15-621)

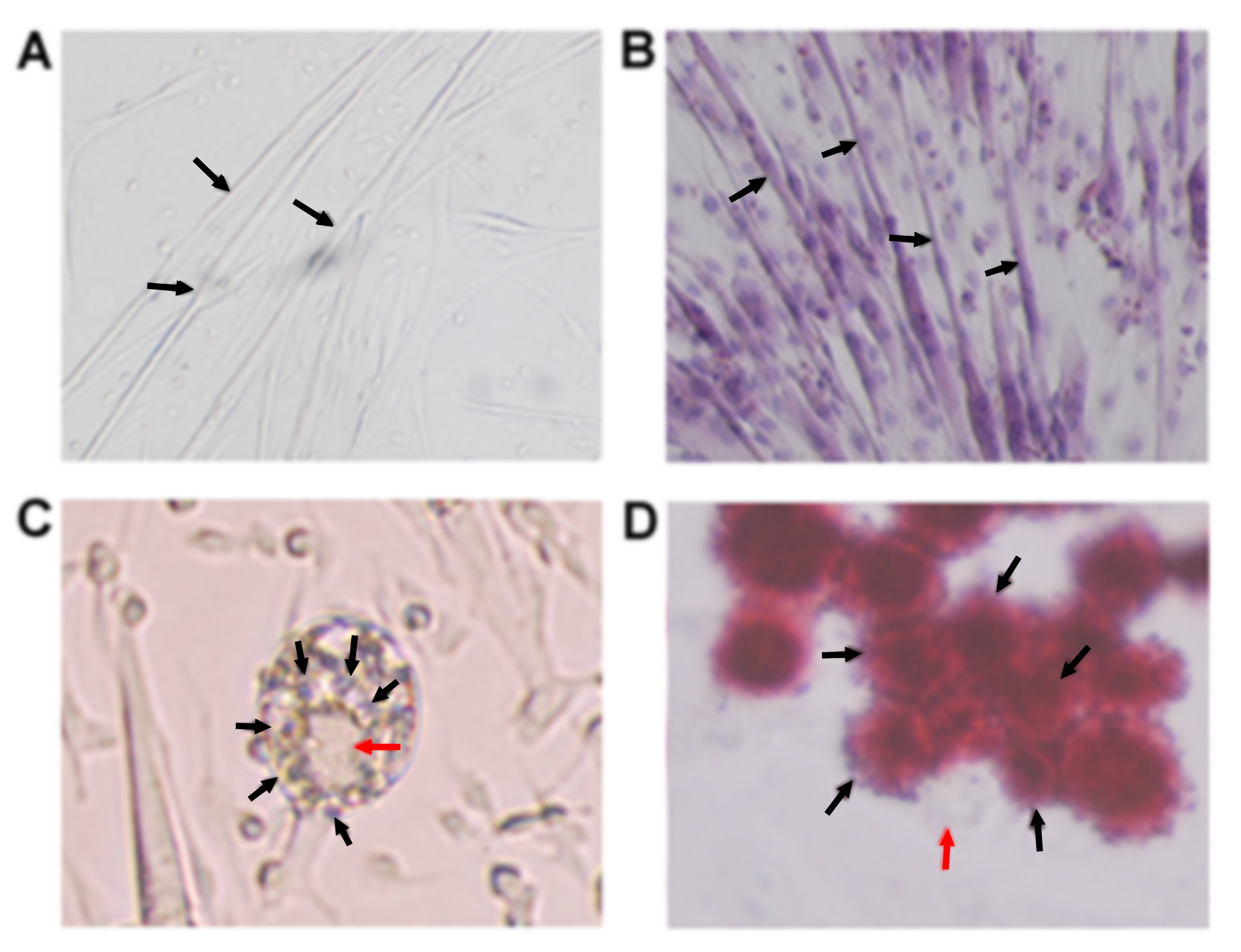

Supplement: Additional file 1 — Satellite cells differentiate into myotubes or early fat storage cells. A, B. Satellite cells at t = 72 h under myogenic differentiation conditions, plated on Matrigel®, without (A) or with (B) hematoxylin/eosin staining. Magnification 100×. Arrows point myotubes. C, D. Satellite cells under early adipogenesis trans-differentiation conditions, plated on Matrigel®, at differentiation time t = 168 h. (C) before and (D) after staining with Oil-Red S. The black arrows show lipid accumulation in cells and red arrow shows the nucleus. Magnification 400×. [file 1471-2164-15-621-S1.zip › 4562148221356466_MOESM1_ESM.bmp]

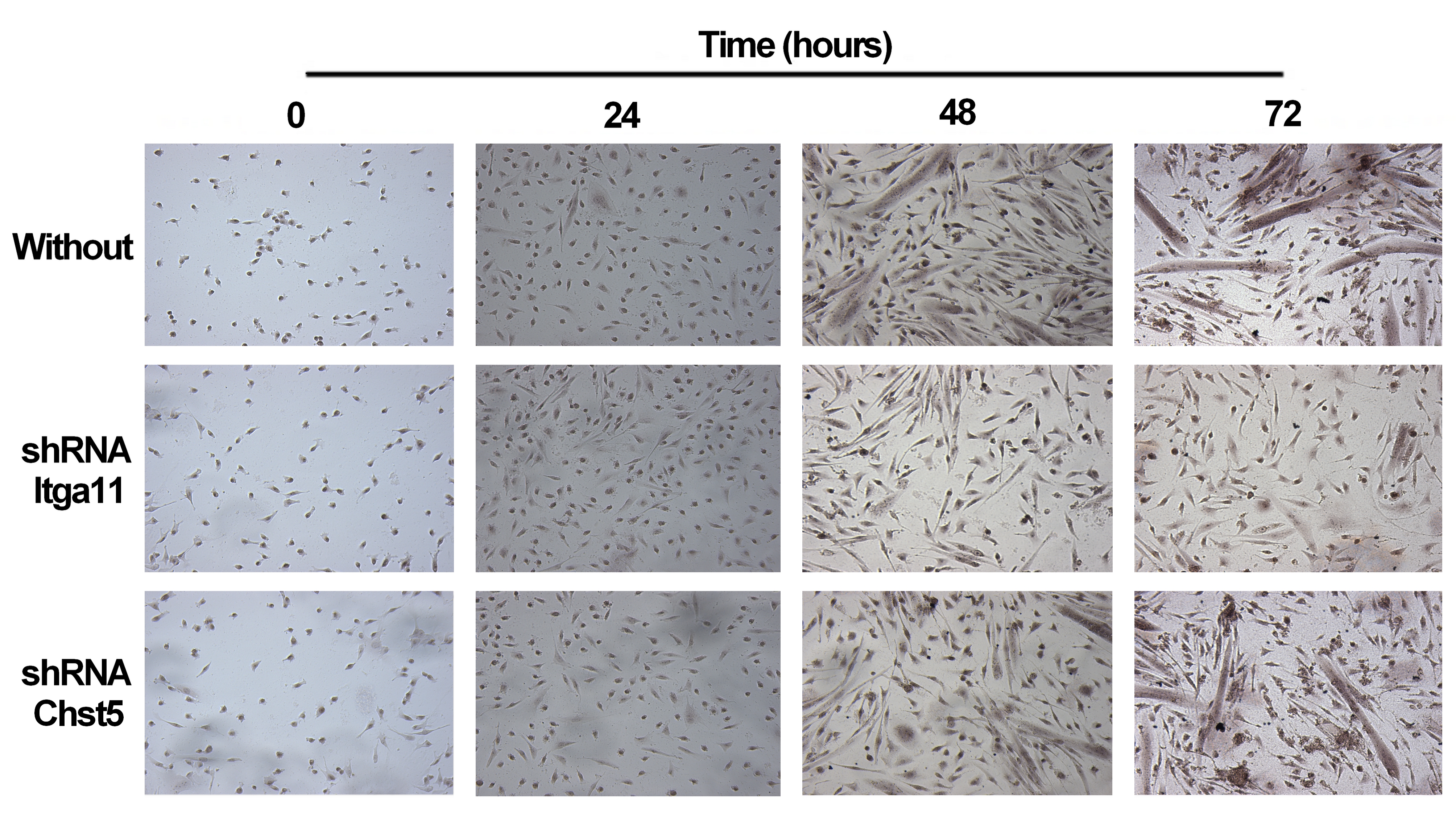

Supplement: Additional file 3 — shRNA against Itga11 and Chst5 inhibit and delayed fusion respectively. Myogenic differentiation of MSC: untreated (Without), treated with plasmid containing shRNA for Itga11 (shRNA Itga11) or shRNA for Chst5 (shRNA Chst5). Photos were taken at different time points after hematoxylin/eosin staining. Magnification was 100×. [file 1471-2164-15-621-S3.jpeg]

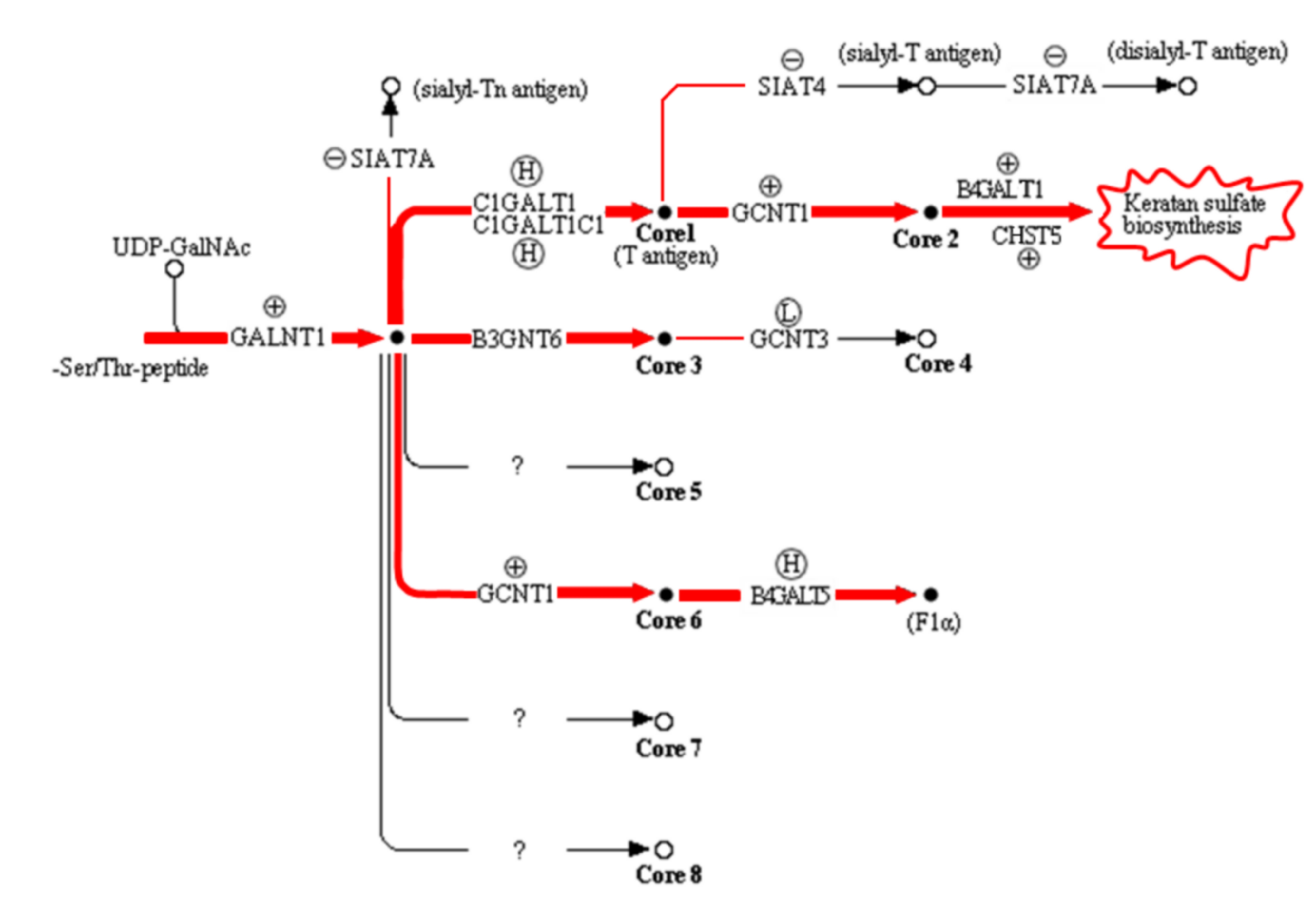

Supplement: Additional file 5 — Orientation of O-glycan biosynthesis. Mucine type O-glycan biosynthetic pathway representation with its enzymes (+: up-regulated, - : down-regulated, H: high constant expression, L: low constant expression) during myogenic differentiation. Red lines symbolize the activated synthetic pathways and the black lines the repressed ones. Core and F1α correspond to the name of the glycan structures: (●) all genes leading to this structure are expressed; (○) some genes in the pathways have no or very low expression. Modified from KEGG Pathway (http://www.genome.jp/kegg/pathway.html). [file 1471-2164-15-621-S5.jpeg]

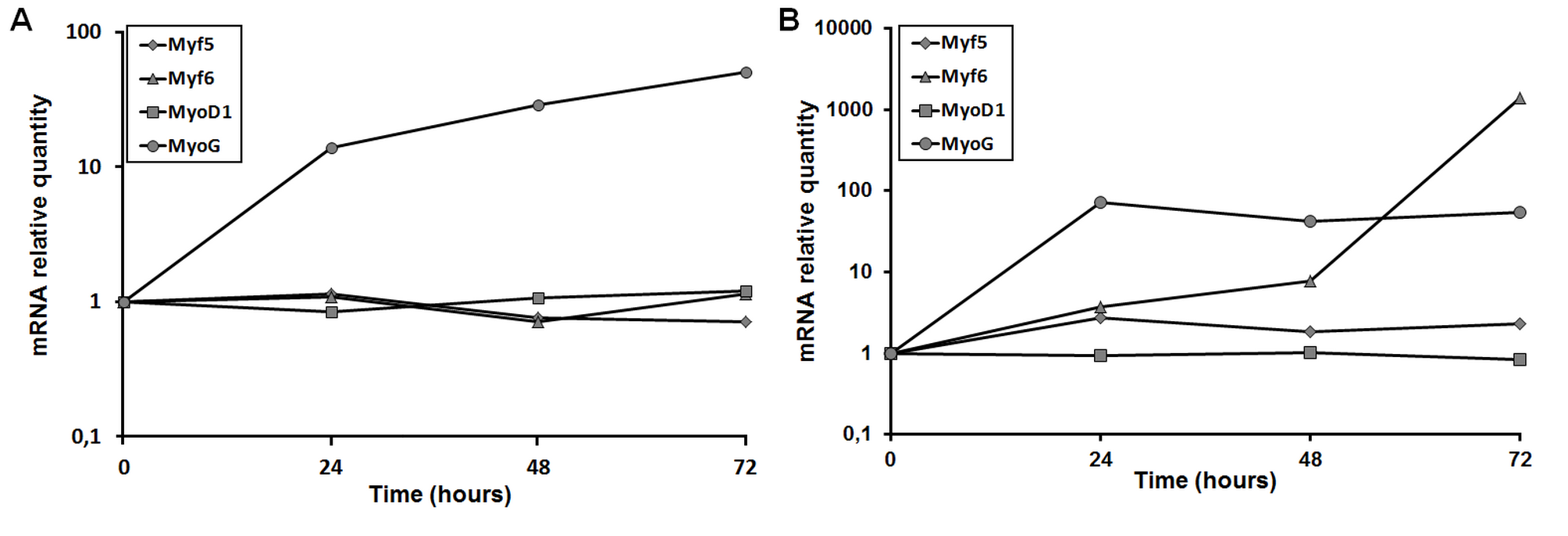

Supplement: Additional file 6 — MRFs expression during differentiation of shRNA-treated cells. A.B. Expression of the MRFs (MyoG (circles), MyoD1 (squares), Myf5 (diamonds), Myf6 (triangles)) during the differentiation of satellite cells treated with shRNA against Itga11 (A) or Chst5 (B). [file 1471-2164-15-621-S6.zip › 4562148221356466_MOESM6_ESM.bmp]
