# Supplementary material for: Highlights of glycosylation and adhesion related genes involved in myogenesis
Source: BMC Genomics. 2014 Jul 22;15:621. doi: 10.1186/1471-2164-15-621 (PMC4223822; doi:10.1186/1471-2164-15-621)
Supplement: Additional file 2 — Sixty-seven genes regulated only during myogenic differentiation of MSC. List of up or down regulated genes during myogenic but not adipogenic MSC differentiation and their expression variation. [file 1471-2164-15-621-S2.pdf]

Gene	Expression pattern
Art1	Up-regulated
Asgr1	Up-regulated
B3galt2	Up-regulated
B4galnt1	Up-regulated
B4galt1	Up-regulated
B4galt4	Up-regulated
Cd248	Up-regulated
Chst12	Up-regulated
Chst5	Up-regulated
Clec2d	Up-regulated
Cmah	Up-regulated
Csgalnact1	Up-regulated
Dpm1	Up-regulated
Fcna	Up-regulated
Galnt2	Up-regulated
Galnt5	Up-regulated
Galntl1	Up-regulated
Gcnt2	Up-regulated
Has1	Up-regulated
Has2	Up-regulated
Hpse	Up-regulated
Icam2	Up-regulated
Idua	Up-regulated
Itga11	Up-regulated
Itga5	Up-regulated
Itga6	Up-regulated
Itga9	Up-regulated
Itgb8	Up-regulated
Itgbl1	Up-regulated
Klra2	Up-regulated
Lctl	Up-regulated
Lgals3bp	Up-regulated
Lgals7	Up-regulated
Lgals9	Up-regulated
Mcam	Up-regulated
Mrc2	Up-regulated
Pigc	Up-regulated
Renbp	Up-regulated
Selp	Up-regulated
Siglecg	Up-regulated
Slc2a10	Up-regulated

Gene	Expression pattern
Chst10	Down-regulated
Chst4	Down-regulated
Chst8	Down-regulated
Clec3b	Down-regulated
Clec4d	Down-regulated
Clgn	Down-regulated
Cplx3	Down-regulated
Fuk	Down-regulated
Fut2	Down-regulated
Fut4	Down-regulated
Fut10	Down-regulated
Gyltl1b	Down-regulated
Hs3st3a1	Down-regulated
Itgb7	Down-regulated
Klrb1a	Down-regulated
Mfng	Down-regulated
Ndst4	Down-regulated
Pitpnm1	Down-regulated
Pmm1	Down-regulated
Sele	Down-regulated
Siglece	Down-regulated
St3gal1	Down-regulated
St3gal5	Down-regulated
St3gal6	Down-regulated
St6galnac2	Down-regulated
St8sia5	Down-regulated
