# Supplementary material for: Highlights of glycosylation and adhesion related genes involved in myogenesis
Source: BMC Genomics. 2014 Jul 22;15:621. doi: 10.1186/1471-2164-15-621 (PMC4223822; doi:10.1186/1471-2164-15-621)
Supplement: Additional file 4 — Three hundred and eighty three genes under study. List of the 383 genes selected from the murine glyco-genome and used for our screening. [file 1471-2164-15-621-S4.pdf]

A	B	C		D - F	G		H	I	
Abo	B3galnt1	C1galt1	Chst4	D4st1	G6pc	Ganc	Has1	Icam2	Itgae
Actb	B3galt1	C1galt1c1	Chst5	Dad1	G6pdx	GAPDH	Has2	Idua	Itgam
Alg11	B3galt2	C76566	Chst7	Ddost	Gaa	Gba	Has3	Itga1	Itgav
Alg12	B3galt4	Calr	Chst8	Dgcr2	Gal3st1	Gba2	Hexa	Itga10	Itgax
Alg1	B3galt5	Calr3	Clec11a	Dpagt1	Galc	Gbgt1	Hexb	Itga11	Itgb1
Alg2	B3galt6	Canx	Clec1b	Dpm1	Galk1	Gcnt2	Hpse	Itga2	Itgb1bp1
Alg3	B3gat1	Cd207	Clec2d	Dpm2	Galnact2	Gcnt3	Hs2st1	Itga2b	Itgb1bp2
Alg5	B3gat2	Cd22	Clec2e	Edem1	Galnt1	Gcs1	Hs3st3a1	Itga3	Itgb2
Alg6	B3gat3	Cd248	Clec2g	Edem2	Galnt10	Ggta1	Hs3st3b1	Itga4	Itgb2l
Alg9	B3gnt1	Cd33	Clec2h	Ext1	Galnt11	Gla	Hs6st2	Itga5	Itgb3
Amy1	B3gnt2	Cd47	Clec2i	Ext2	Galnt12	Glb1	Hs6st3	Itga6	Itgb4bp
Art1	B3gnt3	Cd83	Clec3b	Extl1	Galnt13	Glg1	Hyal1	Itga7	Itgb5
Art2b	B3gnt5	Cd8b1	Clec4a2	Extl2	Galnt2	Glt8d1	Hyal2	Itga8	Itgb6
Art4	B3gnt7	Chi3l1	Clec4b1	Extl3	Galnt3	Glycam1	Hyal3	Itga9	Itgb7
Asgr1	B4galnt1	Chi3l3	Clec4d	Fcna	Galnt4	Gmds		Itgal	Itgb8
Asgr2	B4galnt2	Chi3l4	Clec4e	Fuca1	Galnt5	Gmppa		ItgaD	Itgbl1
Athl1	B4galt1	Chia	Clec4n	Fuca2	Galnt6	Gmppb			
Atrn	B4galt2	Chid1	Clec5a	Fuk	Galnt7	Gne			
	B4galt3	Chpf2	Clec7a	Fut1	Galntl1	Gnpnat1			
	B4galt4	Chst1	Clgn	Fut10	Galntl5	Gpaa1			
	B4galt5	Chst10	Cmah	Fut2	Galt	Gusb			
	B4galt6	Chst11	Cmas	Fut4	Ganab	Gyltl1b			
	B4galt7	Chst12	Cplx3	Fut8					
	Bclp2	Chst2	Csgalnact1						
		Chst3	Ctbs						
			Ctsa						

K	L	M	N	O - P	R	S		T-X
Kl	L1cam	Mag	Naga	Ogt	Renbp	Sec1	Slc5a2	Tbp
Klb	Large	Man1a	Nagk	Olr1	Rfng	Sele	Slc5a3	Tcea1
Klra10	Lctl	Man1a2	Naglu	Parp1	Rft1	Sell	Slc5a4a	Thbd
Klra2	Lfng	Man2a1	Nagpa	Parp2	4930431L04Rik	Selp	Slc5a4b	Tsta3
Klra5	Lgals1	Man2a2	Nans	Parp3	4933434I20Rik	Siglec1	St3Gal1	Uap1
Klra6	Lgals12	Man2b1	Ncam1	Pdia3	Rpn1	Siglece	St3Gal2	Ugcg
Klrb1a	Lgals2	Man2b2	Ncam2	Pecam1	Rpn2	Siglecf	St3Gal3	Ugcgl2
Klrb1c	Lgals3	Man2c1	Ndst1	Pgm1		Siglecg	St3Gal4	Ugdh
Klrc1	Lgals3bp	Manba	Ndst2	Pgm2		Slc2a1	St3Gal5	Ugp2
Klrc2	Lgals7	Manea	Ndst3	Pgm3		Slc2a10	St3Gal6	Ugt2a1
Klrc3	Lgals8	Masp1	Ndst4	Pigb		Slc2a2	St6gal1	Ugt2a3
Klrd1	Lgals9	Masp2	Neu1	Pigc		Slc2a3	St6gal2	Ugt2b1
Klre1	Lman2	Mcam	Neu2	Pigf		Slc2a4	St6GalNac1	Ugt2b34
Klrg1		Mfi2	Neu3	Pigk		Slc2a5	St6GalNac2	Ugt3a2
Klrk1		Mfng	Neu4	Pigm		Slc2a6	St6galnac3	Ugt8
		Mgat2	Ngly1	Pign		Slc2a8	St6GalNac4	Ust
		Mgat3		Pigo		Slc2a9	St6galnac5	Vcam1
		Mgat4a		Pigq		Slc35a1	St6GalNac6	Wbscr17
		Mgat4b		Pigt		Slc35a2	St8sia1	Wdfy3
		Mgat4c		Pitpna		Slc35a3	St8sia2	Xylt1
		Mgat5b		Pitpnb		Slc35b1	St8sia3	Xylt2
		Mgat5		Pitpnm1		Slc35b4	St8sia4	
		Mgea5		Pitpnm2		Slc35c1	St8sia5	
		Mgl1		Pmm1		Slc35d1	St8sia6	
		Mgl2		Pmm2		Slc5a1	Stt3a	
		Mpdu1		Pofut1		Slc5a11	Stt3b	
		Mrc2		Pofut2				
